# Vectorial Proton Transport Mechanism of RxR, a Phylogenetically Distinct and Thermally Stable Microbial Rhodopsin

**DOI:** 10.1038/s41598-019-57122-2

**Published:** 2020-01-14

**Authors:** Keiichi Kojima, Tetsuya Ueta, Tomoyasu Noji, Keisuke Saito, Kanae Kanehara, Susumu Yoshizawa, Hiroshi Ishikita, Yuki Sudo

**Affiliations:** 1 Faculty of Pharmaceutical Sciences, Okayama University, Okayama, 700-8530 Japan; 2 Graduate School of Medicine, Dentistry and Pharmaceutical Sciences, Okayama University, Okayama, 700-8530 Japan; 3 Department of Applied Chemistry, Graduate School of Engineering, The University of Tokyo, Tokyo, 113-8654 Japan; 4 Research Center for Advanced Science and Technology, The University of Tokyo, Tokyo, 153-8904 Japan; 5 Atmosphere and Ocean Research Institute, The University of Tokyo, Chiba, 277-8564 Japan; 6 Department of Natural Environmental Studies, Graduate School of Frontier Sciences, The University of Tokyo, Chiba, 277-8563 Japan

**Keywords:** Biochemistry, Biophysics

## Abstract

*Rubrobacter xylanophilus* rhodopsin (RxR) is a phylogenetically distinct and thermally stable seven-transmembrane protein that functions as a light-driven proton (H^+^) pump with the chromophore retinal. To characterize its vectorial proton transport mechanism, mutational and theoretical investigations were performed for carboxylates in the transmembrane region of RxR and the sequential proton transport steps were revealed as follows: (i) a proton of the retinylidene Schiff base (Lys209) is transferred to the counterion Asp74 upon formation of the blue-shifted M-intermediate in collaboration with Asp205, and simultaneously, a respective proton is released from the proton releasing group (Glu187/Glu197) to the extracellular side, (ii) a proton of Asp85 is transferred to the Schiff base during M-decay, (iii) a proton is taken up from the intracellular side to Asp85 during decay of the red-shifted O-intermediate. This ion transport mechanism of RxR provides valuable information to understand other ion transporters since carboxylates are generally essential for their functions.

## Introduction

Organisms decrease entropy by using external energy to maintain their biological functions. Proton (H^+^) pump proteins exist ubiquitously in cell membranes and produce proton concentration gradients across those membranes by using exergonic energy sources such as the hydrolysis energy of adenosine triphosphate (ATP), reduction-oxidation reactions and light energy^1^. One-directional proton transport is thought to be achieved by the Grotthuss mechanism (i.e., a proton hopping mechanism through several proton-acceptable groups) with continuous p*K*_a_ changes of charged amino acids such as Asp, Glu and Lys, triggered by conformational changes of the protein moiety^2^. Proton gradients across membranes produced by proton pumps play crucial roles in a variety of biological functions such as the maintenance of membrane potential and the incorporation of nutrients into cells^1^.

**Figure 1:**
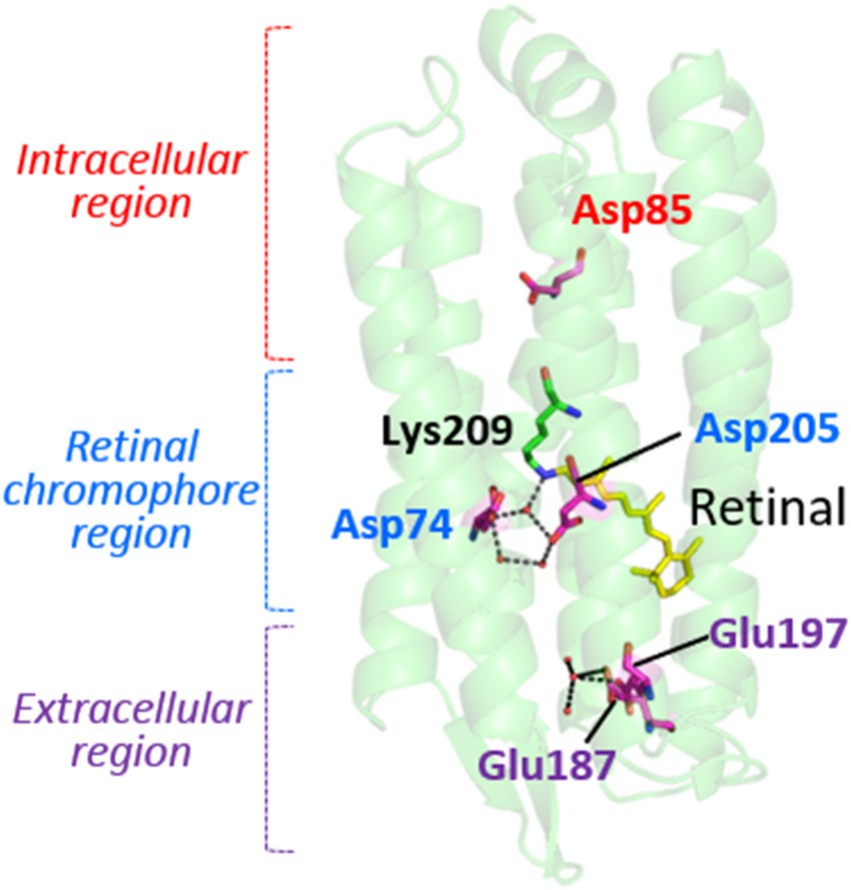
Crystal structure of RxR. The five carboxylates, Schiff base (Lys209), retinal and water molecules are colored magenta, green, yellow and red, respectively.

Microbial rhodopsins consisting of seven-transmembrane α-helices and vitamin-A aldehyde retinal as a chromophore form a large photoreceptor protein family, in which the retinal is bound to a perfectly conserved Lys residue (Lys209 for RxR, see Fig. 1) of the protein moiety “opsin” via a protonated Schiff base linkage^3^. The first microbial rhodopsin, named bacteriorhodopsin (BR), was identified from the halophilic archaeon *Halobacterium salinarum* (formerly *halobium*) as a light-driven outward proton pump in 1971^4^. Since then, several thousands of genes encoding BR-like proton pumps have been widely identified not only from archaea, but also from bacteria and eukarya (Fig. S1A)^5–7^. Genetic, ecological and molecular analyses have shown that proton pump rhodopsins produce comparable amounts of large energy (i.e., ATP) via the formation of proton gradients, indicating the importance of producing proton gradients by rhodopsins for organisms as well as by chlorophyll-based photosynthesis^6^.

The absorption of light by microbial rhodopsins triggers *trans-cis* isomerization of the retinal chromophore, leading to structural changes of opsin through a series of distinct photointermediates, such as the K-, L-, M-, N- and O-intermediates^3,8,9^. During a sequence of reactions called the photocycle, microbial rhodopsins exhibit their photo-dependent biological functions including proton pumps (Fig. S1A). Rhodopsins are an excellent model for understanding ion pumps because of several technical advantages: (i) rhodopsins are activated by light irradiation, and (ii) their activities can be judged by their visible color^3,9^. In 2017, we found a novel proton pump rhodopsin produced by the eubacterium *Rubrobacter xylanophilus*, which lives in a high temperature environment (i.e., 60 °C)^10^. That rhodopsin, named *Rubrobacter xylanophilus* rhodopsin (RxR), is a phylogenetically distinct microbial rhodopsin located between the archaeal and eukaryotic proton pumps (Fig. S1A)^10^, suggesting that RxR can be an excellent model for understanding the relationships between archaeal, eubacterial and eukaryotic proton pump rhodopsins. Moreover, RxR has an extremely high thermal stability^10^ that allowed us to determine its crystal structure at 1.8 Å resolution (PDB code; 6KFQ) (Fig. 1). This study focuses on the analysis of the proton pumping activity and photochemical property of RxR to clarify its vectorial proton transport mechanism. In the structure of RxR, the putative proton transport pathway is roughly divided into three parts, an intracellular region, a retinal chromophore region and an extracellular region (Fig. 1). On the basis of that structural information, in this study we investigated the proton transport mechanism of RxR by combining experimental and computational analyses.

## Results and Discussion

### Photocycle of the wild-type RxR

**Figure 2:**
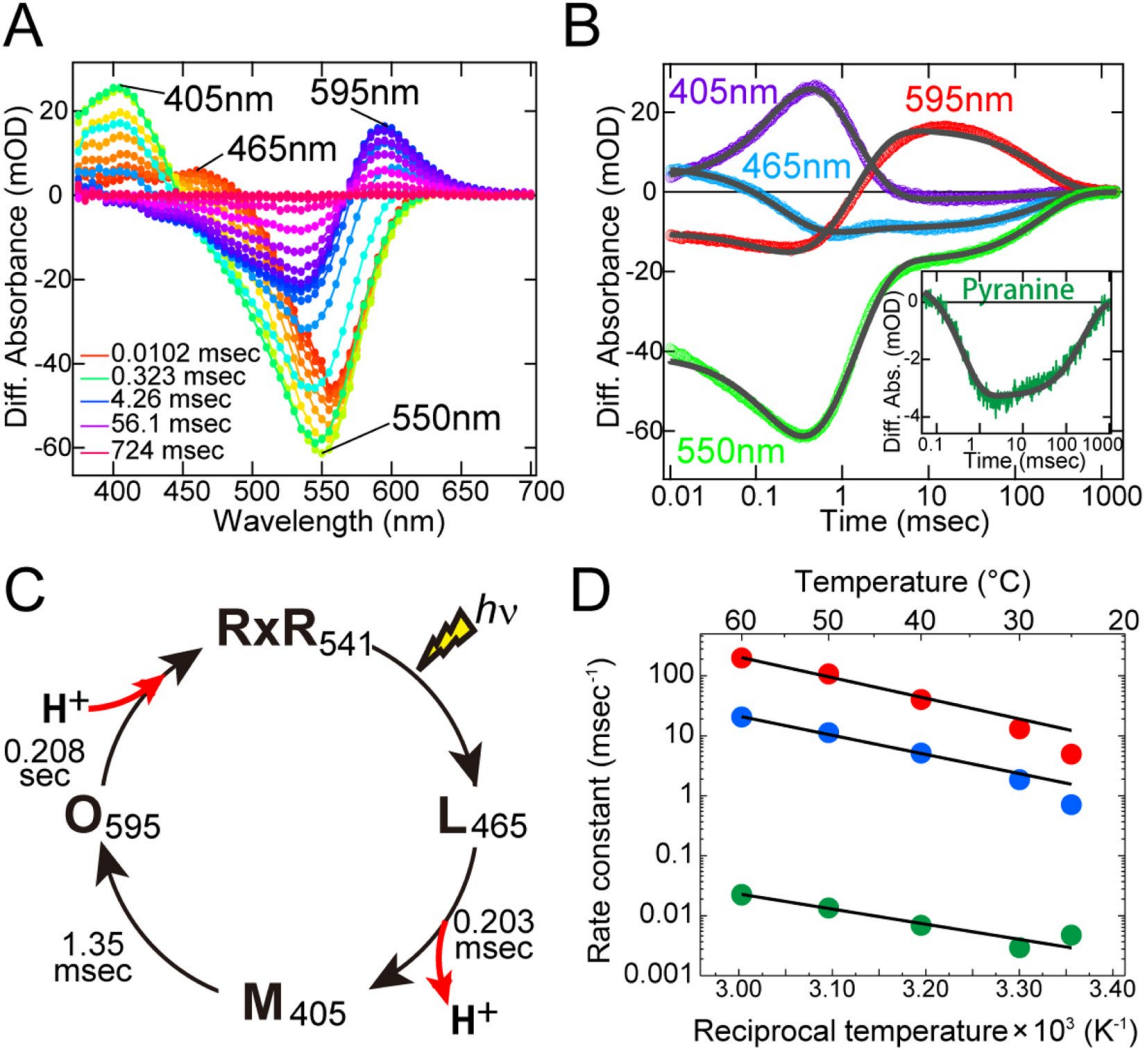
Photocycle of wild-type RxR. (**A**) Time-resolved difference absorption spectra in NaCl solution at 25 °C. (**B**) Time-resolved absorption changes in NaCl solution at 25 °C; the fitting curves are shown as gray. (Inset) Absorption changes of pyranine monitored at 450 nm; the fitting curve is shown as gray. (**C**) Photocycle model with the timing of uptake and release of the proton. (**D**) Arrhenius plots of the rate constants for the transition processes from the L- to M-intermediate (red circles), from the M- to O-intermediate (blue circles), and from the O-intermediate to original state (green circles). The data were analyzed by the Eyring equation to calculate the activation energies (*E*_a_) listed in Table 1. The fitting curves are shown as black lines.

To investigate the photocycle of the wild-type RxR, we performed time-resolved spectroscopic measurements at 25 °C. Figure 2A showed the time-resolved difference spectra of the wild-type RxR. The depletion and recovery at around 550 nm were attributed to changes of the original state (absorption maximum, λ_max_ = 541 nm)^10^. Judging from time region and the location of the absorption maxima, the formation and decay at around 465 nm, 405 nm and 595 nm were attributed to L-, M- and O-intermediates, respectively. Figure 2B showed the time course of the absorption changes at selected wavelengths; 550, 465, 405 and 595 nm for the original state, L-, M- and O-intermediates, respectively. By fitting all the time courses with a triple-exponential function (Fig. 2B, gray curves), we estimated the decay time constants for the L-, M- and O-intermediates as 0.203 (τ_1_), 1.35 (τ_2_) and 2.08 × 10^2^ (τ_3_) msec, respectively (Table S1). Thus, RxR sequentially forms the L-, M- and O-intermediates and then returns to its original state during each photocycle (Fig. 2C). In addition, to clarify the timing of the uptake and release of protons during the photocycle, we monitored the absorption changes of a pH-sensitive dye, pyranine^11^, in the presence and in the absence of RxR. The difference spectrum showed that the pyranine signal decreased within 10 msec (time constant = ~0.4 msec) and then increased within 1,000 msec (time constant = ~2.5 × 10^2^ msec) (Fig. 2B, inset). These time constants coincide well with the M-formation (0.203 msec) and O-decay (2.08 × 10^2^ msec). Because decreases and increases of pyranine signals reflect the acidification and alkalization of the bulk solution, respectively, this result indicates that a proton is first released from RxR upon M-formation and is then taken up from the bulk solution upon O-decay (Fig. 2C). We also investigated the photocycle of the wild-type RxR at varying temperatures. Figure S2 showed the time-resolved difference spectra and the time courses of the absorption changes at 550, 465, 405 and 595 nm for the original state, L-, M- and O-intermediates, respectively, at 30, 40, 50 and 60 °C. The formation and decay of L-, M- and O-intermediates were observed at varying temperatures. By fitting all the time courses of absorption changes with a triple-exponential function (Fig. S2, gray curves), we estimated the rate constants for the transition processes from the L- to M-intermediate (L → M), from the M- to O-intermediate (M → O), and from the O-intermediate to original state (O → original) at 25, 30, 40, 50 and 60 °C (Table S2). Then, to obtain the thermodynamic parameters, the rate constants were plotted against the reciprocal temperature, as shown in Fig. 2D. The plots were analyzed with the Eyring equation as previously described^12^ to calculate the activation energy, *E*_a_. A linear dependence in the Arrhenius plots indicates that each process reflects the transition between two states. The *E*_a_ values for the three transition processes (i.e. L → M, M → O, and O → original) of RxR were estimated to be 63.4, 58.7 and 45.8 kJ/mol, respectively (Table 1), which were higher by 20–26% than those of BR (47, 47 and 36 kJ/mol, respectively)^13^. The high *E*_a_ values for RxR indicate the relatively large temperature dependency on the photocycle of RxR. The rate constants of RxR at physiological temperature (i.e. 60 °C) (L → M, M → O, O → original; 198, 20.6, 2.28 × 10^−2^ msec^−1^, respectively) were similar to those of BR (L → M, M → O, O → original; 200, 2 and 0.2 msec^−1^, respectively)^13^. Thus the high *E*_a_ values make RxR an efficient proton pump at high temperature.

**Table 1:** The activation energies (*E*_a_) for the transition processes from the L- to M-intermediate (L → M), from the M- to O-intermediate (M → O), and from the O-intermediate to original state (O → original) of RxR and those of BR. The *E*_a_ values for BR were subtracted from those for RxR to obtain Δ*E*_a_. The percentages of Δ*E*_a_ to *E*_a_ for RxR were shown in brackets.

	*E*_a_ for RxR	*E*_a_ for BR	Δ*E*_a_
L → M	63.4 kJ/mol	47 kJ/mol^13^	16.4 kJ/mol (26%)
M → O	58.7 kJ/mol	47 kJ/mol^13^	11.7 kJ/mol (20%)
O → original	45.8 kJ/mol	36 kJ/mol^13^	9.8 kJ/mol (21%)

### Mutational effects of carboxylates on proton transport activity

**Figure 3:**
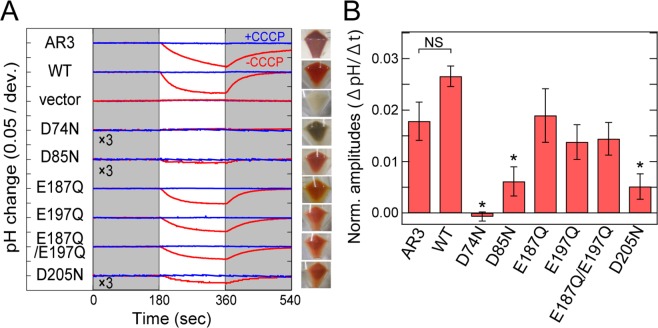
Mutational effects of carboxylates on proton transport activity. (**A**) Light-induced pH changes of suspensions of *E. coli* cells expressing AR3, wild-type RxR and mutants of RxR in the absence or presence of the protonophore, CCCP (red and blue, respectively) in NaCl solution. The suspensions were illuminated with yellow light (>480 nm) for 3 min (white background). As a negative control, *E. coli* cells harboring the pET22b vector were used. Aligned images on the right show *E. coli* cells expressing AR3, wild-type RxR and mutants of RxR. The amplitudes of pH changes for D74N, D85N and D205N were enlarged by 3 times for the comparison. (**B**) Quantitative evaluation of the proton pumping activities of AR3, wild-type RxR and mutants of RxR. The initial slope amplitudes of the light-induced pH changes in NaCl solution, which were obtained from the data in panel A, were normalized against the total amounts of photoactive proteins (Fig. S4). All error bars represent standard error of the mean (SEM) of three independent measurements (n = 3). Asterisks (*) indicate a significant difference from wild-type RxR (P < 0.05; Dunnett’s test). NS indicates no significant difference (P > 0.05; Dunnett’s test).

It is known that, not only for retinal-based ion pumps, but also for other ion pumps, carboxylates play essential roles in their cognate ion transport^3,8,14–16^. The secondary structure of RxR contains several well-conserved carboxylates in its transmembrane domains, i.e. Asp74, Asp85, Glu187, Glu197 and Asp205 (Figs. 1 and S1B)^10^. To elucidate the roles of those carboxylates, we constructed and analyzed their neutralized mutants (i.e., D74N, D85N, E187Q, E197Q, D205N and E187Q/E197Q). Wild-type and mutant RxR proteins were expressed as recombinant proteins in *Escherichia coli* cells. As a positive control, a homologous proton pump rhodopsin to BR, Archaerhodopsin-3 (AR3)^17^, was expressed in *E. coli* cells. Then we checked the colors of those cells to confirm the expression of the proteins as “holoproteins”. The visible colors of the cells of all RxR mutants indicated their successful expression as holoproteins in *E. coli* cells. The cells of all mutants except for D74N showed red color, whereas D74N showed brownish color (Fig. 3A), suggesting that the absorption maximum of D74N was shifted to the longer wavelength. Of note, from the colors of the cells, we can judge only the expression of the holoproteins, but not the amplitude of their proton pumping activities. Then, to investigate the proton pumping activities, we measured the light-induced pH changes of the *E. coli* cell suspensions. While the significant light-induced pH changes of RxR were observed at 25 °C as previously described^10^, no significant changes were observed at physiological temperature (i.e. 60 °C) (Fig. S3A), which implies that the proton pumping activity cannot be quantitatively analyzed at 60 °C probably because *E. coli* cells were lysed by heat. Therefore, we analyzed the activities at 25 °C. As shown in Fig. 3A, light-induced decreases in environmental pH were observed for the wild-type, D85N, E187Q, E197Q, E187Q/E197Q and D205N RxR proteins as well as AR3, implying an outward proton movement from the intracellular to the extracellular side of the cells upon illumination, while the decreases in pH were impaired by the presence of the protonophore carbonyl cyanide *m*-chlorophenylhydrazone (CCCP). The amplitude of light-induced pH decrease for the wild-type RxR was approximately 0.1 unit, which seems to be lower than that for BR (approx. 0.25–1 unit) in purple membrane and liposomes^18–20^. However experimental conditions were different between RxR and BR, therefore it is hard to judge whether the proton pumping activity of RxR is lower or not as compared with BR. Of note, these data of Fig. 3A indicate that the five RxR mutant proteins, D85N, E187Q, E197Q, E187Q/E197Q and D205N, maintain their proton pump function, although their signal amplitudes are lower than wild-type RxR. On the other hand, detectable signals were not observed for D74N with or without CCCP as well as a negative control (vector), suggesting that D74N lacks proton pumping activity.

In order to quantitatively estimate proton pumping activities, we obtained the initial slope amplitudes of extracellular pH changes from 0 to 10 sec after illumination in the absence of CCCP^21,22^. We used 15 mW/cm^2^ as a light intensity because the relationship between light intensities and initial slope amplitudes of pH changes showed a linear regression at light intensities below 104 mW/cm^2^ (see the text of Experimental procedures for details) (Fig. S3B). In addition, we also assumed that the proton pumping activity depends on the protein expression level in the cells. Therefore, the total amounts of photoactive proteins were calculated by estimating the absorbance derived from holoproteins in the visible light region^23^ (see the text of Experimental procedures for details) (Fig. S4). The initial slope amplitudes were then normalized by the total amounts of the photoactive holoproteins (Fig. 3B). The normalized amplitude of wild-type RxR was 1.5-fold larger than that of AR3, but the difference was not significant, suggesting that the proton pumping activity of RxR is comparable with that of AR3 in *E. coli* cells. The amplitudes of E187Q, E197Q and E187Q/E197Q were 0.64-, 0.45- and 0.55-fold lower, respectively, than wild-type RxR, but the differences were not significant. On the other hand, the amplitudes of D85N and D205N were significantly lower than wild-type RxR (0.26- and 0.24-fold for D85N and D205N, respectively), indicating that Asp85 and Asp205 are involved in the proton pumping function. Of note, the amplitude of D74N was strongly impaired to almost zero, indicating that Asp74 is essential for proton pump function. To further investigate the role of the five carboxylates during the proton pumping process, we performed spectroscopic analysis of the purified RxR proteins. For many microbial rhodopsins, it is known that the corresponding mutant of D205N is rapidly denatured during the purification process in detergent because of its low stability, while fortunately, all RxR mutants including D205N were successfully purified without significant denaturation. This would be due to the high thermal stability of RxR^10^.

### Role of Asp74 and Asp205

**Figure 4:**
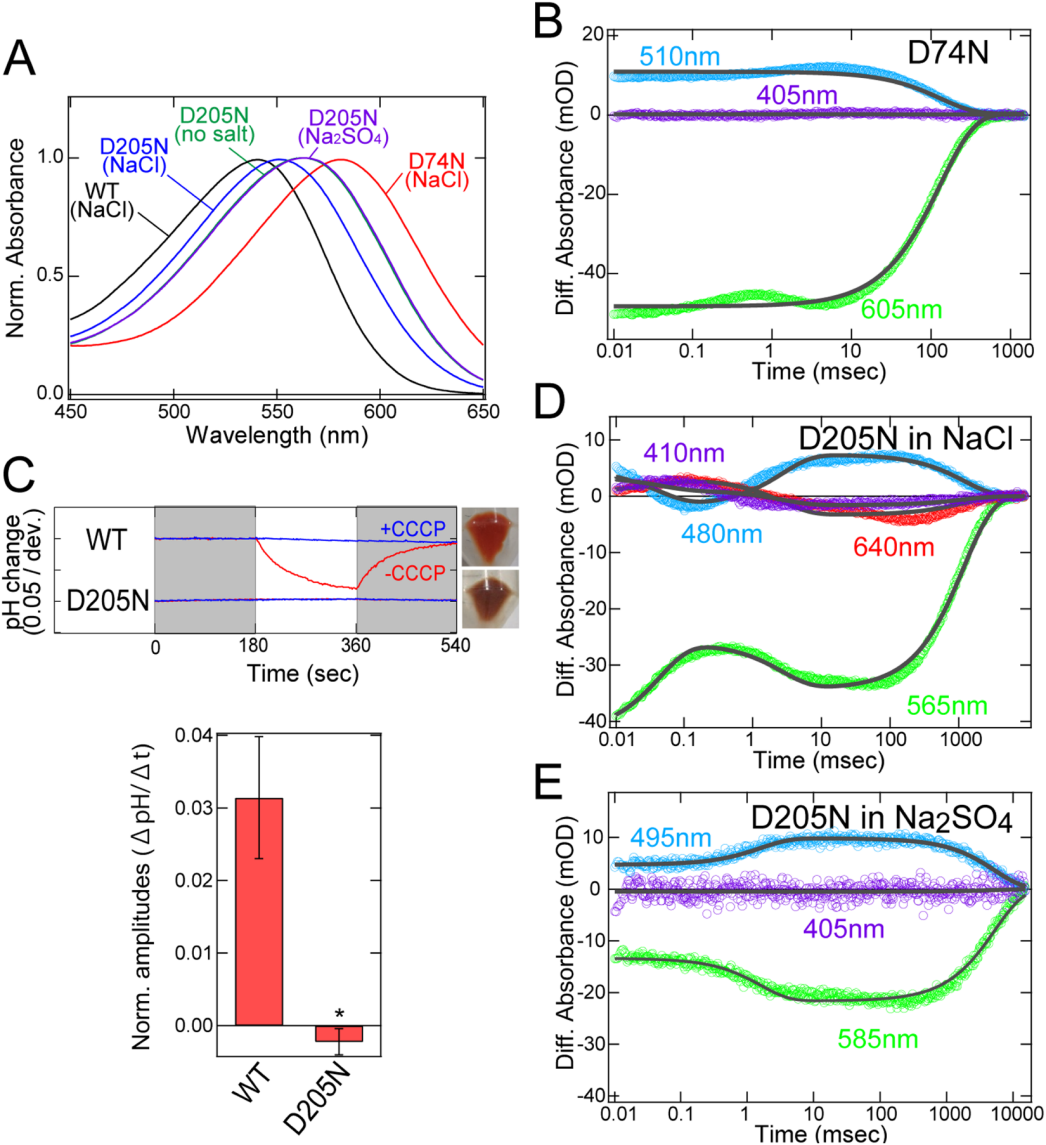
Mutational effects of Asp74 and Asp205 located around the retinal chromophore. (**A**) Comparison of absorption spectra of wild-type RxR and the D74N and D205N RxR mutants in detergent n-dodecyl-β-D-maltoside (DDM) micelles. Absorption spectra of wild-type RxR and D74N were measured in NaCl solution, whereas those of D205N were measured in NaCl, in Na_2_SO_4_ and in no salt solutions. All spectra were normalized at peak absorbance. (**B**) Time-resolved absorption changes of D75N in NaCl solution at 25 °C; the fitting curve is shown as gray. (**C**) (upper panel) Light-induced pH changes of suspensions of *E. coli* cells expressing wild-type RxR and D205N in the absence or presence of the protonophore, CCCP (red and blue, respectively) in Na_2_SO_4_ solution. The suspensions were illuminated with yellow light (>480 nm) for 3 min (white background). Images on the right show *E. coli* cells expressing wild-type RxR and D205N. (lower panel) Quantitative evaluation of the proton pumping activities of wild-type RxR and D205N in Na_2_SO_4_ solution. The initial slope amplitudes of the light-induced pH changes, which were obtained from the data in Fig. S4, were normalized against the total amounts of photoactive proteins. All error bars represent the SEM of three independent measurements (n = 3). An asterisk (*) indicates a significant difference from the wild-type RxR (P < 0.05; Dunnett’s test). (**D,E**) Time-resolved absorption changes of D205N in NaCl (**D**) and in Na_2_SO_4_ solution (**E**); the fitting curves are shown as gray.

Regarding the role of the retinal chromophore region, we focused on Asp74 and Asp205. The crystal structure of RxR indicated that Asp74 and Asp205 form a pentagonal hydrogen-bonding network with water molecules near the protonated Schiff base (Fig. 1). To estimate the protonation states of the carboxylates at the original (unphotolyzed) state, we calculated p*K*_a_ values (hereafter calculated p*K*_a_). The calculated p*K*_a_ values of 2.8 for Asp74 and 0.2 for Asp205 suggest that Asp74 and Asp205 are deprotonated (Table S3). It should be noted that the calculated p*K*_a_ values of the corresponding carboxylates of BR (e.g., 1.5 for Asp85 and −2.0 for Asp212^24^) are consistent with the experimentally determined values (e.g., 2.6 for Asp85^25^ and <2.5 for Asp212^26^) (Table S3). Absorption spectra of wild-type RxR and the D74N and D205N RxR mutant proteins in NaCl solution are shown in Fig. 4A. The absorption maximum (λ_max_) of D74N (581 nm) was largely red-shifted compared with wild-type RxR (541 nm) (Table S1). Neutralization of the negatively charged counterion of the Schiff base in microbial rhodopsins decreases the energy gap between the electronic ground and excited states of the retinal, leading to the spectral red shift^27^. Thus, the spectral red shift of D74N indicates that the negatively charged Asp74 stabilizes the protonated Schiff base as a counterion. In proton pump rhodopsins, a substrate proton is transported through several proton-acceptable groups including carboxylates during the transition process of several photointermediates. To investigate the role of Asp74 on the proton transportation, we analyzed the photocycle of D74N. Upon illumination, D74N showed the formation and decay of only a photointermediate at around 510 nm (Figs. 4B and S5). By fitting the time course with a single-exponential function (Fig. 4B, gray curves), we estimated the decay time constant for the photointermediate as 130 (τ_1_) msec (Table S1). In contrast to the photocycle of wild-type RxR, the M-intermediate was not observed in the photocycle of D74N. From the analogy with BR^3,8,9^, we estimated that the deprotonated Asp74 accepts a substrate proton from the retinylidene Schiff base upon formation of the M-intermediate. From these results, the lack of proton pumping activity of D74N is explained by the lack of the M-intermediate.

We then characterized the D205N mutant. The λ_max_ of D205N (551 nm) was also red-shifted compared with wild-type RxR (541 nm) in NaCl solution (Fig. 4A). That difference in λ_max_ indicates that Asp205 is not protonated in wild-type RxR and that the negative charge of the deprotonated Asp205 stabilizes the protonated Schiff base as a secondary counterion. Of note, the spectral red shift of D205N (Δ = 10 nm) is much smaller than that of D74N (Δ = 40 nm), which implies that the neutralization effect of Asp205 is partial and that some polar molecule, such as Cl^−^ and H_2_O, would be a surrogate of a negatively charged Asp205 residue. To test that hypothesis, we measured the absorption spectra of D205N in Cl^−^ free solutions. For wild-type RxR, the absorption spectra in NaCl, Na_2_SO_4_ and no salt solutions were totally overlapped (Fig. S6A and Table S1). In contrast, while the absorption spectra of D205N in Na_2_SO_4_ and no salt solutions were totally overlapped among them, that in Na_2_SO_4_ solution showed a ~17 nm spectral red-shift compared to that in the NaCl solution, indicating Cl^–^ binding to D205N (Fig. 4A and Table S1). To elucidate the Cl^–^binding effect on the proton pumping function, we also measured the light-induced pH changes in Cl^–^free Na_2_SO_4_ solution (Fig. 4C). The signals of wild-type RxR in Na_2_SO_4_ solution were comparable with those in NaCl solution (Figs. 3B and 4C). In contrast, D205N did not show a detectable pH change in Na_2_SO_4_ solution (Fig. 4C), indicating that D205N lacks proton pumping activity in Na_2_SO_4_ solution. Thus, in addition to Asp74, Asp205 is also essential for the proton pumping function. To clarify its mechanism, we measured the photocycle of D205N in NaCl and in Na_2_SO_4_ solutions (Figs. 4D,E and S5). For wild-type RxR, the overall photocycles were almost identical in NaCl and Na_2_SO_4_ solutions (Figs. 2A,B, S6 and Table S1), while, for D205N, an M-like photointermediate was formed at around 410 nm in NaCl but not in Na_2_SO_4_ solution (Figs. 4D,E and S5). Thus, the lack of the M-intermediate in Na_2_SO_4_ solution corresponds to the lack of proton pumping activity of D205N in Na_2_SO_4_ solution (Fig. 4C). Additionally, the proton pumping activity of D205N is partially rescued by binding to Cl^−^, but it is significantly lower than that of wild-type RxR (Figs. 3B, 4A,C). By fitting the time courses of the absorption changes at 410, 480, 565 and 640 nm with a triple-exponential function (Fig. 4D, gray curves), we estimated the three time constants (τ_1_, τ_2_, τ_3_) as shown in Table S1. The time constant of the rate-limiting step in the photocycle of D205N in NaCl (τ_3_ = 1.19 × 10^3^ msec) is approximately 6-fold larger than that of wild-type RxR (τ_3_ = 2.08 × 10^2^ msec), suggesting that the low proton pumping activity of D205N with Cl^−^ (0.24-fold) is due to its slow photocycle with Cl^−^.

### Role of Asp85

**Figure 5:**
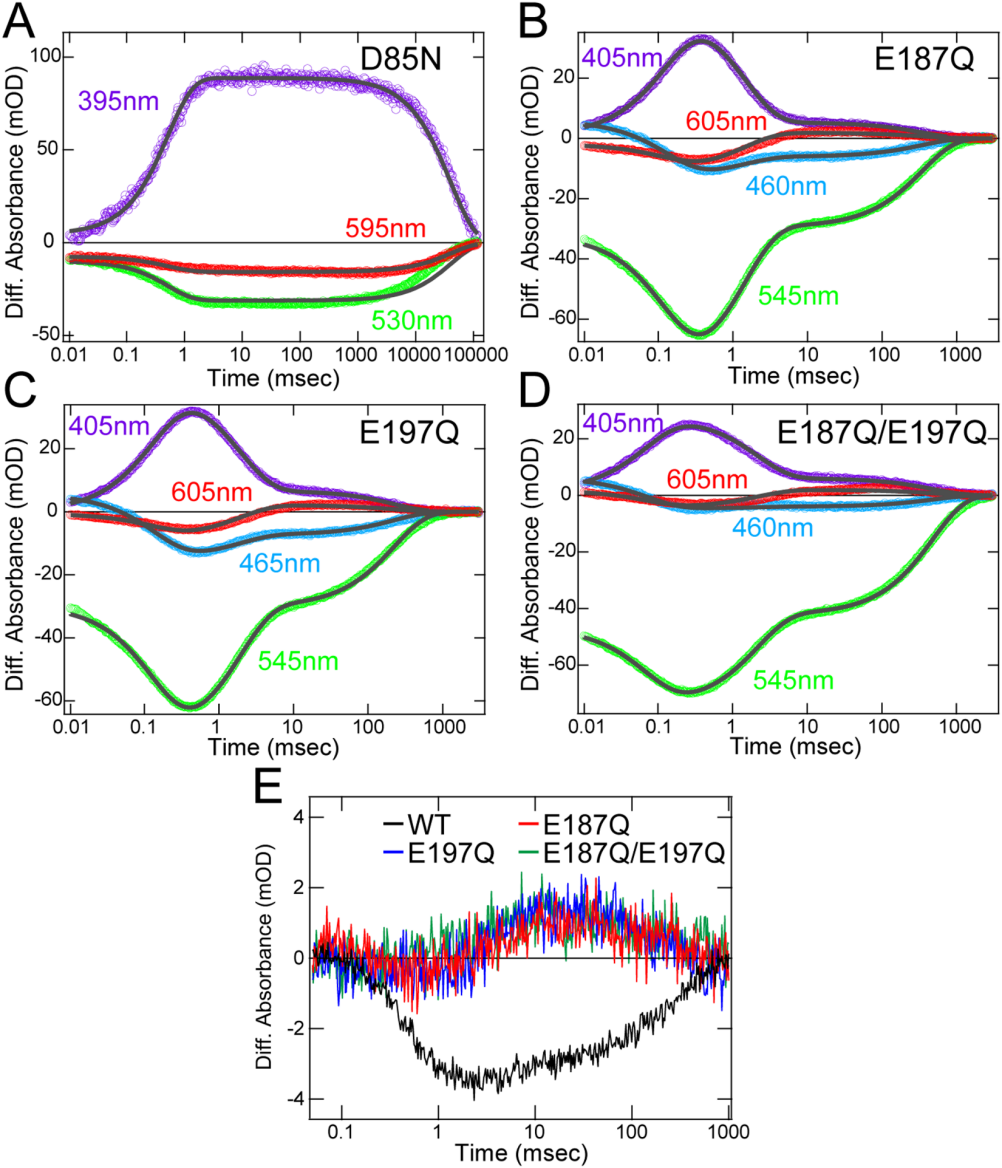
Mutational effects of Asp85 and Glu187/Glu197 located in the intracellular and extracellular sides of RxR, respectively. (**A**) Time-resolved absorption changes of D85N in NaCl solution at 25 °C; the fitting curves are shown as gray. (**B**–**D**) Time-resolved absorption changes of E187Q (**B**), E197Q (**C**) and E187Q/E197Q (**D**) in NaCl solution at 25 °C. (**E**) Absorption changes of pyranine monitored at 450 nm of the wild-type RxR, and the E187Q, E197Q and E187Q/E197Q RxR mutants at 25 °C. The absorption changes of E187Q, E197Q and E187Q/E197Q were enlarged 4 times for comparison.

To understand the role of the intracellular region, we focused on Asp85. As mentioned above, the proton pumping activity of D85N was 0.26-fold lower than the wild-type RxR (Fig. 3B), suggesting that Asp85 contributes to substrate proton transport probably during the process of proton uptake because the proton comes from the intracellular side. The λ_max_ of D85N (542 nm) is almost identical to that of wild-type RxR (541 nm) (Fig. S7 and Table S1), which suggests that this mutation on the intracellular side does not affect the structure around the retinal chromophore. To investigate the role of Asp85 on the proton transportation, we analyzed the photocycle of D85N. Upon illumination, D85N formed an M-like photointermediate at around 395 nm (Figs. 5A and S5). By fitting the time courses of the absorption changes at 395, 530 and 595 nm with a double-exponential function (Fig. 5A, gray curves), we estimated the formation and decay time constants for the M-intermediate as 0.520 msec (τ_1_) and 40.2 sec (τ_2_), respectively (Table S1). As well as D205N in NaCl solution, the slow photocycle would lead to the lower proton pumping activity of D85N (Fig. 3B). Of note, the decay rate of the M-intermediate in D85N is ~200-fold slower than in wild-type RxR. Thus, the deficiency of Asp85 inhibits the efficient substrate proton transfer to the Schiff base, suggesting that Asp85 works as a proton donor to the Schiff base during M-decay. Asp85 is located in the highly hydrophobic intracellular side, which is likely to be protonated. Consistently, the calculated p*K*_a_ value of Asp85 is 11.9, which is much higher than neutral pH (Table S3). Together with the photocycle model of RxR shown in Fig. 2C, we conclude that protonated Asp85 transfers a proton to the Schiff base during M-decay and is reprotonated by proton uptake from the intracellular side during O-decay.

### Role of Glu187 and Glu197

To understand the role of the extracellular region, we focused on Glu187 and Glu197. As mentioned above, the proton pumping activity of E187Q, E197Q and E187Q/E197Q were 0.64-, 0.45- and 0.55-fold lower, respectively, than wild-type RxR, but those differences were not significant (Fig. 3B). The crystal structure of RxR showed that Glu187 and Glu197 form a unique hydrogen-bonding network with water molecules in the extracellular side (Fig. 1), suggesting that Glu187 and Glu197 play important roles in the process of proton release. To investigate the roles of Glu187 and Glu197 on the proton transportation, we analyzed the photocycle of the mutants. Upon illumination, E187Q, E197Q and E187Q/E197Q showed sequential formation of the three photointermediates, L, M and O, at ~465, 405 and 595 nm, respectively, with the breaching of the original state at 545 nm similar to wild-type RxR (Figs. 5B–D and S5). By fitting the time courses of the absorption changes with a triple-exponential function (Fig. 5B–D, gray curves), we estimated the decay time constants for the L-, M- and O-intermediates (τ_1_, τ_2_, τ_3_, respectively) in the three mutants as shown in Table S1. The decay time constants in the three mutants and in wild-type RxR were observed in the same time frames (Table S1). Thus, the similar photocycle could explain the similar proton pumping activities among them (Fig. 3B). The λ_max_ of E187Q, E197Q and E187Q/E197Q (538, 538 and 537 nm, respectively) are almost identical to wild-type RxR (541 nm) (Fig. S7 and Table S1), which suggests that these mutations on the extracellular side of RxR do not affect the structure around the retinal chromophore. To investigate the effects of E187Q and E197Q mutations on the proton releasing process, we monitored the absorbance changes of pyranine in the three RxR mutants (Fig. 5E). The absorption of pyranine increased within 30 msec and then decreased within 1000 msec in the three RxR mutants. These signals indicate that a proton is firstly taken up from the bulk solution and is then released from the proteins in the three RxR mutants. Thus, the substitutions of Glu187 and Glu197 inverted the order of proton release and uptake, strongly suggesting that both Glu187 and Glu197 form a proton releasing group at the extracellular side in wild-type RxR. The calculated p*K*_a_ values of 8.3 for Glu187 and 5.7 for Glu197 (Table S3) indicate that Glu187 is protonated while Glu197 is deprotonated in the hydrogen-bonding network at neutral pH. Therefore, a proton would be released from Glu187. Of note, not only the substitution of Glu187, but also that of Glu197 inverted the order of proton release and uptake. To explain that fact, we assumed that the deprotonated Glu197 maintains the protonation state of Glu187 in the hydrogen-bonding network. Together with the photocycle model of RxR shown in Fig. 2C, we conclude that a proton is released from the releasing group consisting of Glu187, Glu197 and water molecules during M-formation and the releasing group is reprotonated probably during O-decay.

### Vectorical proton transport mechanism of RxR

**Figure 6:**
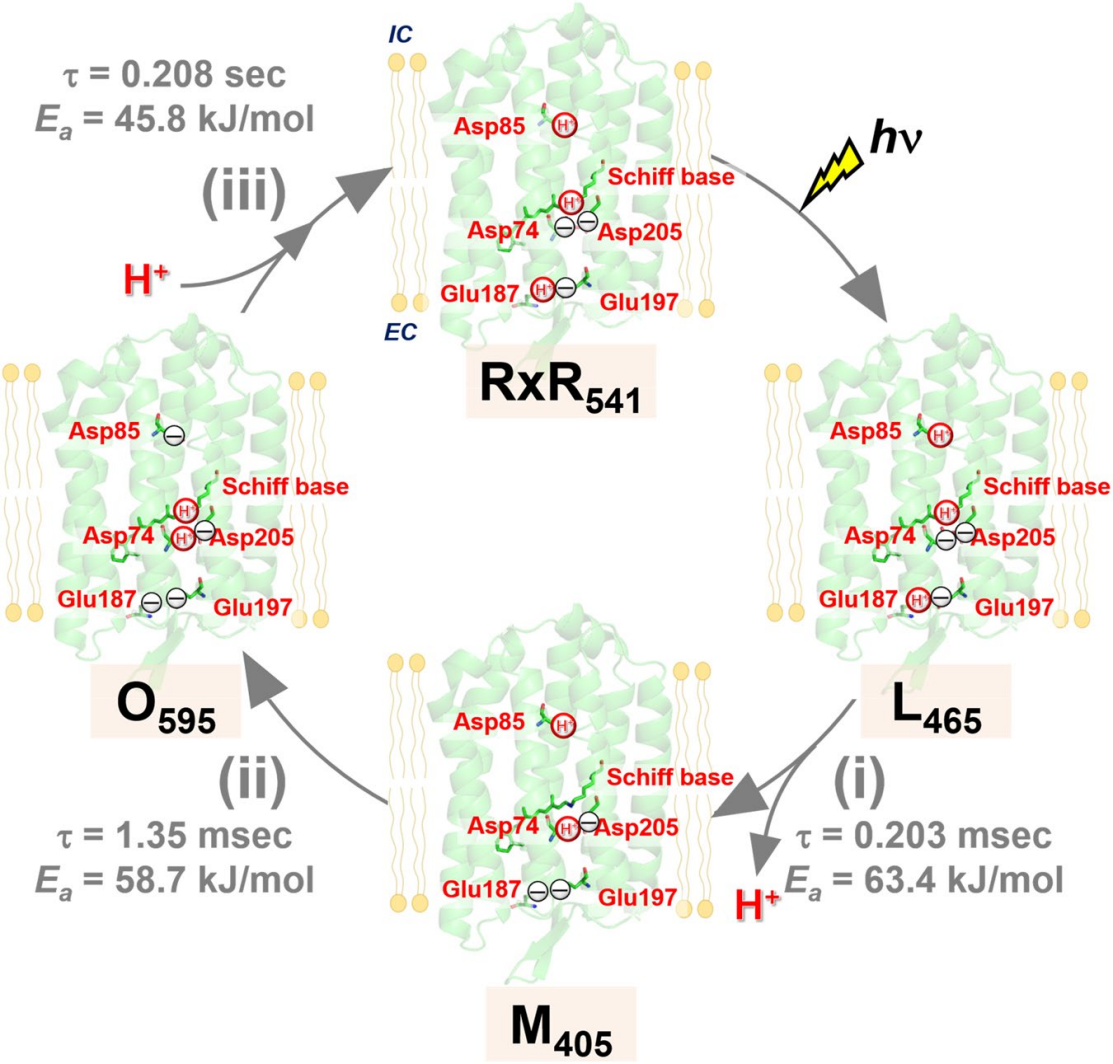
Molecular mechanism of vectorial proton transport in RxR. A proton is transported from the intracellular side to the extracellular side during a single photoreaction cycle through several carboxylates. The protonation/deprotonation states of the five carboxylates in the original state (RxR_541_) and the L-, M- and O-intermediates (L_465_, M_405_ and O_595_, respectively) are shown. EC and IC indicate extracellular and intracellular sides, respectively.

To understand the vectorial proton transport mechanism of RxR, we would like to have brief comparison with that of a prototypical proton pump rhodopsin BR. In BR, Asp85 (Asp74 in RxR) and Asp96 (Asp85 in RxR) work as a proton acceptor from the protonated Schiff base in collaboration with Asp212 (Asp205 in RxR), and as a proton donor to the deprotonated Schiff base, respectively^3^. Glu194/Glu204 (Glu187/Glu197 in RxR) form the hydrogen-bonding network with water molecules as a proton releasing group^3^. Based on the background, we propose the sequential steps of proton transport of RxR (shown schematically in Fig. 6). In the original state (RxR_541_ in Fig. 6), Asp85, Glu187 and the Schiff base are protonated, whereas Asp74, Glu197 and Asp205 are deprotonated. The absorption of light triggers *trans*-*cis* isomerization of retinal and it leads to the formation of the L-intermediate. During the transition from the L- to M-intermediate (the process (i) in Fig. 6), a proton of the Schiff base is transferred to the counterion Asp74 in collaboration with the negatively charged Asp205, leading to the deprotonation and protonation of the Schiff base and Asp74, respectively. Simultaneously with the above step, a proton is released from the proton releasing group (Glu187/Glu197) to the extracellular side, leading to the deprotonation of Glu187. Then, during the transition from the M- to O-intermediate (the process (ii) in Fig. 6), a proton of Asp85 is transferred to the Schiff base, leading to the deprotonation and protonation of Asp85 and the Schiff base, respectively. Finally, during the transition from the O-intermediate to the original state (the process (iii) in Fig. 6), a proton is taken up from the intracellular side to Asp85, leading to the protonation of Asp85. As a result, a proton is transported from the intracellular side to the extracellular side during a single photocycle. Thus, we successfully determined the proton transport mechanism of RxR in detail. So far, as a model not only for proton pumps but also for membrane proteins, the microbial rhodopsin BR has been widely analyzed using physicochemical methods, such as high-speed atomic force microscopy (AFM)^28^ and x-ray free-electron laser (XFEL)^29^ analysis from the perspective of physical chemistry. It should be noted that the thermal stability of RxR is ~200-fold greater than BR in detergent micelles^10^. Thus, among microbial rhodopsins, including BR, RxR could be the most suitable model for comprehensive physicochemical analysis, particularly methods that require high protein stability. Moreover, it is known that carboxylates play essential roles for ion transport in various ion transporters^14–16^, however, the comprehensive roles of carboxylates are still unclear in many cases. Considering that the functional importance of carboxylates is common among various ion transporters, the proton transport mechanism of RxR, in which carboxylates compose the ion transport pathway across the molecule and substrate ions are hopping by the rational control of their p*K*_a_ values, would be applicable to other various ion transporters.

## Methods

### Gene preparation, protein expression and purification

The *Escherichia coli* strains, DH5α and BL21(DE3), were used as hosts for DNA manipulation and for protein expression, respectively. An expression plasmid of histidine-tagged RxR was constructed as previously described^10^ and RxR mutant genes were constructed by the SLiCE method^30^ using the gene of histidine-tagged RxR as a template. Consequently, these genes were inserted into pET21a (for wild-type RxR) or pET22b (for all RxR mutants) plasmid vectors (Novagen, USA) with NdeI and XhoI restriction enzyme sites. An expression plasmid of histidine-tagged Archaerhodopsin-3 (AR3) was constructed as previously described^22^. *E. coli* cells harboring the plasmids were grown at 37 °C in LB medium (1% Bacto tryptone, 0.5% yeast extract and 0.5% NaCl) containing 50 μg/mL ampicillin and protein expression was induced by the addition of isopropyl β-D-1-thiogalactopyranoside (IPTG, final conc. = 1 mM) and all-*trans* retinal (final conc. = 10 μM). The cells were disrupted by sonication and crude membrane fractions were solubilized with n-dodecyl-β-D-maltoside (DDM, DOJINDO, Japan, 1.5 w/v %) and then purified by Ni^2+^ affinity column chromatography against the histidine-tag. Purified proteins were concentrated by centrifugation using an Amicon Ultra filter (30,000 MW cutoff; Merck Millipore, USA) and then replaced by the appropriate buffer solution.

### Quantitative analysis of proton pumping activities

Proton transport activity was measured by light-induced environmental pH changes of *E. coli* cell suspensions using essentially the same method as previously described^10,31^. In short, *E. coli* cells were grown at 37 °C in LB medium containing 50 μg/mL ampicillin until the optical density at 660 nm reached to 0.4–0.6. After that, the cells were incubated at 37 °C for 3 hr with IPTG (final conc. = 1 mM) for RxR or L(+)-arabinose (final conc. = 0.1% (w/v)) for AR3, and all-*trans* retinal (final conc. = 10 μM). The cells were then collected by centrifugation and washed three times in 0.3 M NaCl or 0.1 M Na_2_SO_4_ solutions. After that, the cells were kept in the dark for 10 min and were illuminated with a Xenon lamp through a cut-off filter (>480 nm), where the light intensity was adjusted to 15 mW/cm^2^ at 550 nm using an optical power meter. Light-induced pH changes were monitored using a pH electrode in the presence or absence of the protonophore carbonyl cyanide *m*-chlorophenylhydrazone (CCCP, final conc. = 40 μM). The temperature of the samples was maintained at 25 °C using a thermostat. The initial slope amplitudes of the light-induced pH changes from 0 to 10 sec after light irradiation were used as the proton pumping activity of RxR and AR3 proteins^21,31^.

After the measurements, the cells were collected by centrifugation (5,535 × *g* for 10 min) and then resuspended in a buffer solution containing 50 mM Tris-HCl (pH 7.0) and 300 mM NaCl. The cells were then disrupted by sonication in ice-cold water to decrease the scatter of the samples for the spectroscopic measurements. The membrane fraction was collected by ultracentrifugation (178,203 × *g* for 30 min). Absorption spectra were measured using a UV-visible spectrophotometer (UV2450, Shimadzu, Japan) with an ISR2200 integrating sphere (Shimadzu, Japan) at room temperature (approx. 25 °C). To estimate the total amounts of photoactive proteins, the absorption spectra were mathematically deconvoluted (Fig. S4)^23^. Firstly, the contribution of background light scattering to the spectra was fitted by the power functions of reciprocal wavelength (λ) such that ${\rm{\alpha }}+{({\rm{\beta }}/{\rm{\lambda }})}^{\gamma }$. Secondly, the residual spectra were fitted with four log-normal equations that are composed of three vibronic bands (p1, p2 and p3) of the chromophore (p1; the main band, p2; the unknown band, p3; the denatured band) and an additional band (p4) for the UV absorption of the protein. The log-normal equation is as follows;

1 $$A(\lambda )=A\times {e}^{-\frac{ln2}{(\mathrm{ln}\rho )}}\times [ln(\frac{(\frac{1}{\lambda }\,-\,\frac{1}{{\lambda }_{max}})({\rho }^{2}-1)}{\rho \,\times \omega })]$$

where λ_max_ is wavelength (nm); ω is half-bandwidth (cm^−1^); ρ is parameter of skewness; A(λ) is absorbance; A is amplitude as previously described^32^. From the absorbance of the main band (p1) at the wavelength of λ_max_ and its molecular extinction coefficient (ε = 54,000 for RxR and ε = 64,828 for AR3)^10,33^, the total amounts of photoactive proteins were estimated. Finally, the proton pumping activities of wild-type RxR, AR3 and RxR mutants were normalized against the amounts of photoactive proteins.

### Absorption spectra of purified samples

Absorption spectra of purified samples were recorded with a UV-visible spectrophotometer (UV2450 or UV1800, Shimadzu, Japan). The samples were suspended in a buffer (0.89 mM citric acid, 0.89 mM MES, 1.1 mM TES, 0.78 mM TAPS, 1.1 mM CHES, 0.33 mM CAPS (pH 7.0) and 0.05% DDM) containing 1 M NaCl, 333 mM Na_2_SO_4_ or no salt.

### Time-resolved spectroscopy

The apparatus and the procedure for time-resolved flash-photolysis experiments were essentially the same as previously described^34^. In short, transient time-resolved absorption spectra from 375 to 700 nm at 5 nm intervals were measured at time intervals of 0.5 μs using a homemade computer controlled flash photolysis system with a Nd:YAG laser as an actinic light source. The wavelengths of the actinic pulse were tuned using an optical parametric oscillator, where the pulse intensity was adjusted to 2 mJ per pulse. The temperature of the sample was kept at 25, 30, 40, 50 and 60 °C using a thermostat. The experiments were performed in buffer (50 mM Tris-HCl (pH 7.0), 0.05% DDM) containing 0.3 M NaCl or 0.1 M Na_2_SO_4_. To monitor proton uptake and release during the photocycle, the pH indicator pyranine (final conc. = 100 μM) was used as previously described^34^. The absorption changes of pyranine were measured using unbuffered samples in a solution containing 0.3 M NaCl, 0.05% DDM to enhance the signals.

### Protonation pattern and pK_a_ of the carboxylates in RxR and in BR

As a basis for the computations, the crystal structures of BR (PDB code; 1C3W^35^) and RxR (PDB code; 6KFQ) were used. H atoms were generated and energetically optimized with CHARMM22^36^, and all titratable groups were kept in their standard protonation states. Atomic partial charges of the amino acids were adopted from the all-atom CHARMM22^36^ parameter set.

To obtain p*K*_a_ values in the protein environment, we calculated the electrostatic energy difference between the protonated and deprotonated states in a reference model system by solving the linear Poisson-Boltzmann equation with the MEAD program^37^. The present computation is based on the electrostatic continuum model created by solving the linear Poisson-Boltzmann equation with the MEAD program^37^. To facilitate a direct comparison with previous computational results^24^, identical computational conditions and parameters, such as atomic partial charges and dielectric constants, were used. To obtain absolute p*K*_a_ values of target sites (e.g., p*K*_a_(Asp85) of BR), we calculated the difference in electrostatic energy between the two protonation states, protonated and deprotonated, in a reference model system using a known experimentally measured p*K*_a_ value (e.g., 4.0 for Asp^38^). The difference in the p*K*_a_ value of the protein relative to the reference system was added to the known reference p*K*_a_ value. The experimentally measured p*K*_a_ values employed as references were 7.2 for the Schiff base^39,40^, 12.0 for Arg, 4.0 for Asp, 9.5 for Cys, 4.4 for Glu, 10.4 for Lys, 9.6 for Tyr^38^, and 7.0 and 6.6 for the Nε and Nδ atoms of His, respectively^41–43^. All other titratable sites were fully equilibrated to the protonation state of the target site during the titration. The ensemble of the protonation patterns was sampled by a Monte Carlo method with Karlsberg^44^. The dielectric constants were set to *ε*_*p*_* = *4 inside the protein and *ε*_*w*_ = 80 for water. All computations were performed at 300 K, pH 7.0, and an ionic strength of 100 mM. The linear Poisson-Boltzmann equation was solved using a three-step grid-focusing procedure at resolutions of 2.5 Å, 1.0 Å and 0.3 Å. The MC sampling yielded the probabilities [protonated] and [deprotonated] of the two protonation states of the molecule. The p*K*_a_ value was evaluated using the Henderson-Hasselbalch equation. A bias potential was applied to obtain an equal amount of both protonation states ([protonated] = [deprotonated]), yielding the p*K*_a_ value as the resulting bias potential.

## Supplementary information

Supplemetary information.
