# Supplementary material for: Vectorial Proton Transport Mechanism of RxR, a Phylogenetically Distinct and Thermally Stable Microbial Rhodopsin
Source: Sci Rep. 2020 Jan 14;10:282. doi: 10.1038/s41598-019-57122-2 (PMC6959264; doi:10.1038/s41598-019-57122-2)
Supplement: Supplementary file 1 — Supplemetary information. [file 41598_2019_57122_MOESM1_ESM.docx]

**Supporting Information**

**Vectorial Proton Transport Mechanism of RxR, a Phylogenetically Distinct and Thermally Stable Microbial Rhodopsin**

Keiichi Kojima^1,2,#^, Tetsuya Ueta^2,#^, Tomoyasu Noji^3,4^, Keisuke Saito^3,4^, Kanae Kanehara^1^, Susumu Yoshizawa^5,6^, Hiroshi Ishikita^3,4^ & Yuki Sudo^1,2*^

^1^Faculty of Pharmaceutical Sciences, Okayama University, Okayama 700-8530, Japan; ^2^Graduate School of Medicine, Dentistry and Pharmaceutical Sciences, Okayama University, Okayama 700-8530, Japan; ^3^Department of Applied Chemistry, Graduate School of Engineering, The University of Tokyo, Tokyo 113-8654, Japan; ^4^Research Center for Advanced Science and Technology, The University of Tokyo, Tokyo 153-8904, Japan; ^5^Atmosphere and Ocean Research Institute, The University of Tokyo, Chiba 277-8564, Japan; ^6^Department of Natural Environmental Studies, Graduate School of Frontier Sciences, The University of Tokyo, Chiba 277-8563, Japan.

^#^These authors contributed equally to this work.

*To whom correspondence should be addressed: Yuki Sudo: Graduate School of Medicine, Dentistry and Pharmaceutical Sciences, Okayama University, Okayama 700-8530, Japan; [sudo@okayama-u.ac.jp](mailto:sudo@okayama-u.ac.jp); Tel: +81-86-251-7945.

**Table S1.** Photochemical properties of wild-type RxR and mutants of RxR at 25 °C

| Opsin type | Absorption maxima | Time constants in the photocycle |
| --- | --- | --- |
| WT | 541 nm (NaCl)  540 nm (Na_2_SO_4_)  540 nm (no salt) | τ_1_, τ_2_, τ_3_ = 0.203, 1.35, 208 msec (NaCl)  τ_1_, τ_2_, τ_3_ = 0.185, 1.03, 407 msec (Na_2_SO_4_) |
| D74N | 581 nm (NaCl) | τ_1_= 130 msec (NaCl) |
| D85N | 542 nm (NaCl) | τ_1_, τ_2_ = 0.520, 4.02×10^4^ msec (NaCl) |
| E187Q | 538 nm (NaCl) | τ_1_, τ_2_, τ_3_ = 0.137, 1.48, 345 msec (NaCl) |
| E197Q | 538 nm (NaCl) | τ_1_, τ_2_, τ_3_ = 0.143, 1.97, 220 msec (NaCl) |
| E187Q/E197Q | 537 nm (NaCl) | τ_1_, τ_2_, τ_3_ = 0.0748, 2.17, 483 msec (NaCl) |
| D205N | 551 nm (NaCl)  562 nm (Na_2_SO_4_)  562 nm (no salt) | τ_1_, τ_2_, τ_3_ = 0.0449, 2.72, 1.19 ×10^3^ msec (NaCl)  τ_1_, τ_2_ = 1.60, 5.31×10^3^ msec (Na_2_SO_4_) |

**Table S2.** The rate constants for the transition processes from the L- to M-intermediate (k_1_), from the M- to O-intermediate (k_2_), and from the O-intermediate to original state (k_3_) of wild-type RxR at 25, 30, 40, 50 and 60 °C

| Temperature | Rate constants in the photocycle |
| --- | --- |
| 25 °C | k_1_, k_2_, k_3_ = 4.94, 0.710, 4.77×10^-3^ msec^-1^ |
| 30 °C | k_1_, k_2_, k_3_ = 13.1, 1.85, 2.93×10^-3^ msec^-1^ |
| 40 °C | k_1_, k_2_, k_3_ = 40.2, 5.15, 6.92×10^-3^ msec^-1^ |
| 50 °C | k_1_, k_2_, k_3_ = 107, 11.3, 1.34×10^-2^ msec^-1^ |
| 60 °C | k_1_, k_2_, k_3_ = 198, 20.6, 2.28×10^-2^ msec^-1^ |

**Table S3.** Experimental (Exp.) and calculated (Calc.) p*K*_a_ values of the Schiff base and carboxylates

| Opsin type | Schiff base | Primary counterion | Secondary counterion | Proton donor | Proton releasing group | Ref. |
| --- | --- | --- | --- | --- | --- | --- |
| RxR | Exp. ; 10.7^[a]^ (Lys209) | Exp. ; 1.3^[a]^  Calc. ; 2.8  (Asp74) | Calc. ; 0.2  (Asp205) | Calc. ; 11.9  (Asp85) | Calc. ; 8.3 (Glu187)  Calc. ; 5.7 (Glu197) | This study  [a]: ^1^ |
| BR | Exp. ; 13.3^[b]^  (Lys216) | Exp. ; 2.6^[c]^  Calc. ; 1.5^[f]^  (Asp85) | Exp. ; <2.5^[d,e]^  Calc. ; -2.0^[f]^  (Asp212) | Exp. ; >12.0^[d,e]^  Calc. ; 10.9  (Asp96) | Calc. ; 10.4 (Glu194)  Calc. ; 3.6 (Glu204) | [b]: ^2^  [c]: ^3^  [d]: ^4^  [e]: ^5^  [f]: ^6^ |


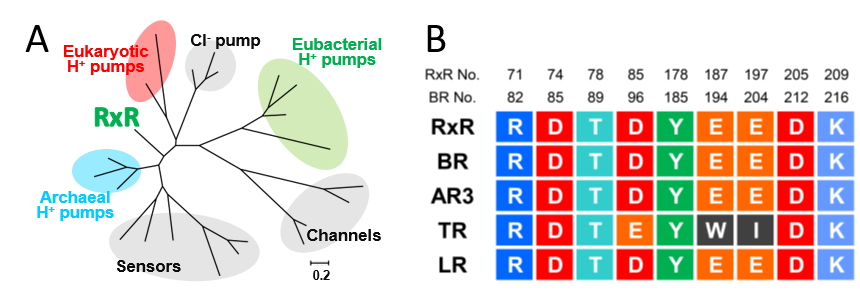


**Figure S1. Phylogenetic location and conserved residues of RxR.**

(A) Phylogenetic tree of microbial rhodopsins. Unrooted maximum likelihood tree of microbial rhodopsin amino acid sequences. Amino acid sequences of microbial rhodopsins including RxR were aligned using MUSCLE, and evolutionary distances were estimated using the JTT matrix-based method.^7^ (B) Sequence alignment of amino acid residues known to be important for proton transport in proton pump rhodopsins. RxR, BR, AR3, LR and TR indicate *Rubrobacter xylanophilus* rhodopsin (Genbank accession number: ABG04982), bacteriorhodopsin from *Halobacterium salinarum* (AAG19772), archaerhodopsin-3 from *Halorubrum sodomense* (BAA09452), *Leptosphaeria* rhodopsin (AAG01180) and thermophilic rhodopsin (AFH39233), respectively. RxR and TR have been identified from the eubacterial domain, AR3 and BR have been identified from the archaeal domain, while LR has been identified from the eukaryotic domain.


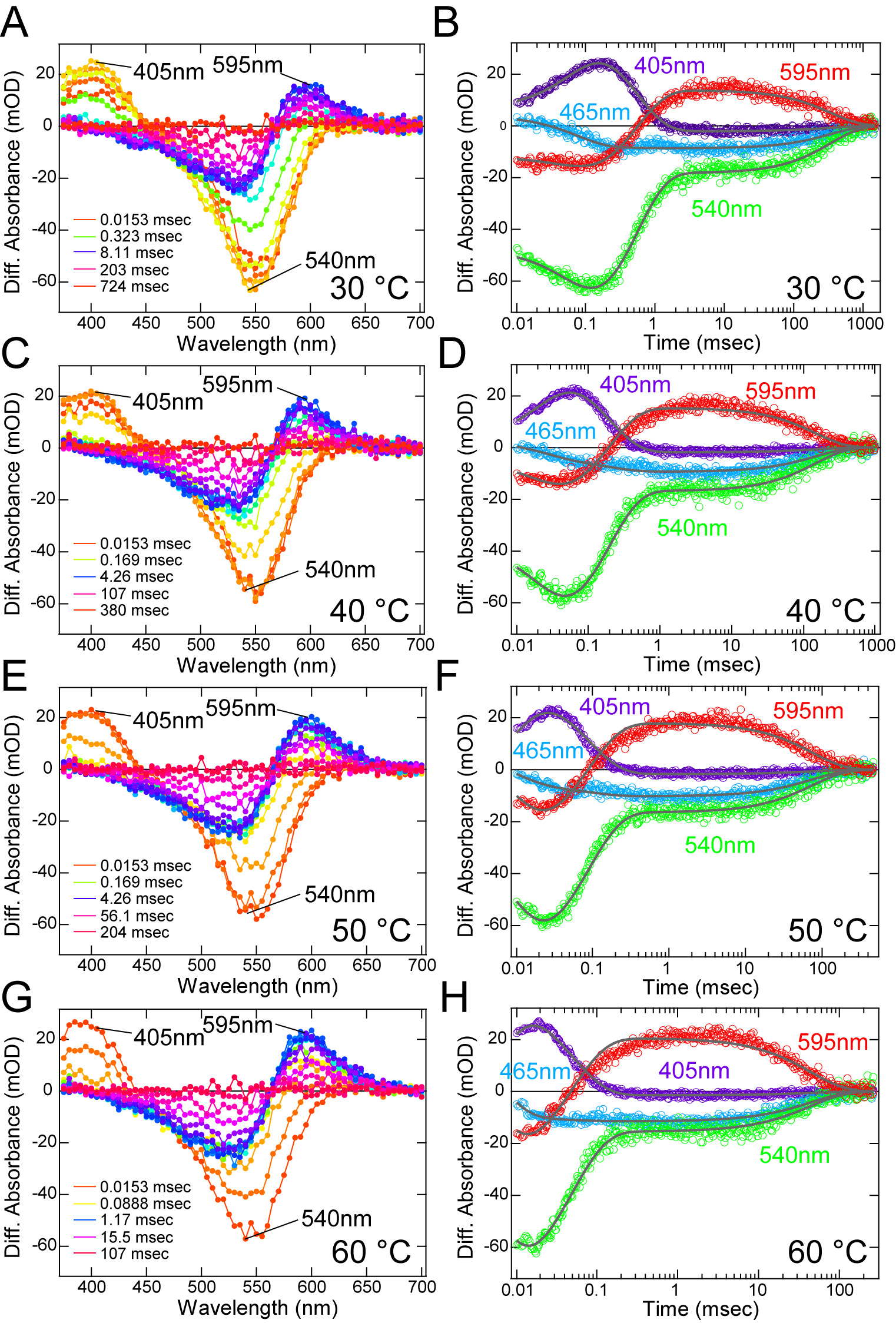


**Figure S2. Temperature-induced changes of photoreaction of wild-type RxR.**

Flash-photolysis analysis in NaCl solution at 30, 40, 50 and 60 °C. Time-resolved difference absorption spectra a spectral range from 375 to 700 nm at 30 (A), 40 (C), 50 (E) and 60 °C (G). Time-resolved absorption changes at 30 (B), 40 (D), 50 (F) and 60 °C (H).


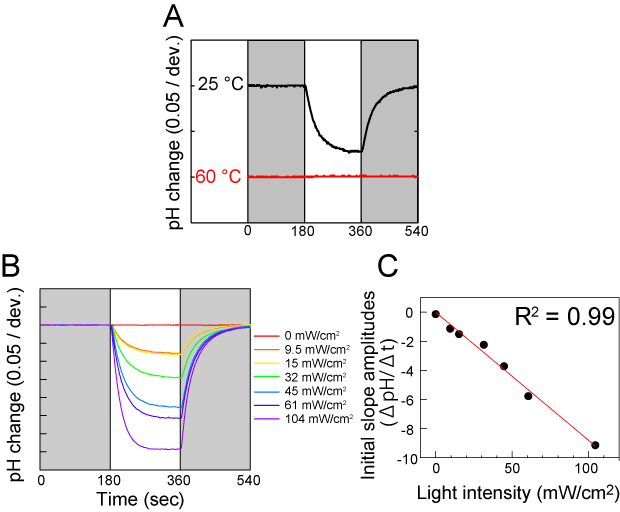


**Figure S3. Temperature and intensity dependence of light-induced pH changes for suspensions of *E. coli* cells expressing wild-type RxR.**

(A) Temperature-dependent light-induced pH changes of suspensions of *E. coli* cells expressing wild-type RxR in NaCl solution at 25 and 60 °C. The suspensions were illuminated with yellow light (> 480 nm) for 3 min (white background). (B) Intensity-dependent pH changes of suspensions of *E. coli* cells expressing wild-type RxR in NaCl solution at 25 °C. The intensity was varied from 0 to 104 mW/cm^2^. The suspensions were illuminated with yellow light (> 480 nm) for 3 min (white background). (C) Relationship between the initial slope amplitudes and light intensities. The amplitudes of initial slope amplitudes of the light-induced pH changes (panel B) during 10 sec after illumination were plotted against varying intensities of light. The data were fitted well by a linear regression (R^2^ = 0.99).


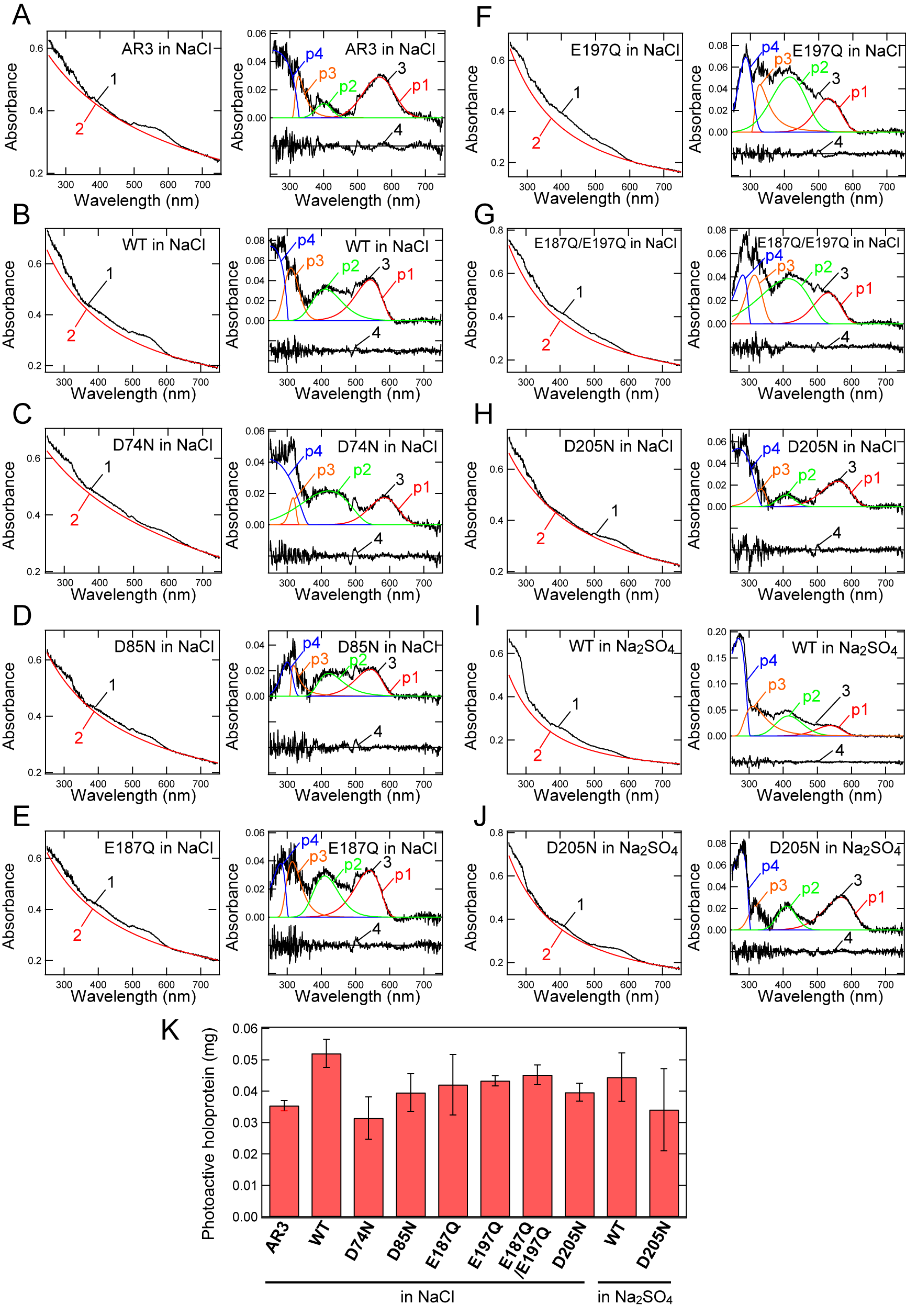


**Figure S4. Quantitative estimation of the amounts of photoactive proteins in *E. coli* cells.**

(A - J) Estimation of the total amounts of photoactive proteins in *E. coli* cells expressing AR3 (A), wild-type RxR (B), or RxR mutants, D74N (C), D85N (D), E187Q (E), E197Q (F), E187Q/E197Q (G) and D205N (H) in NaCl solution and wild-type RxR (I) and D205N (J) in Na_2_SO_4_ solution. Curve 1 is the absorption spectrum of the suspension of cell membranes. The contribution of the background light scattering (curve 2) was subtracted from curve 1 to obtain the spectrum consisting of the absorption of photoactive proteins and contaminated proteins (curve 3). Curve 3 was fitted by four log-normal equations (p1, p2, p3 and p4). Curve 4 is the residual spectrum between curve 3 and the fitting curve. From the absorbance of the main band (p1) at the wavelength of λ_max_, the total amounts of photoactive proteins were estimated. (K) Comparison of the total amounts of photoactive holoproteins in *E. coli* cells expressing AR3, wild-type RxR and mutants of RxR. All error bars represent the SEM of three independent measurements (n = 3).


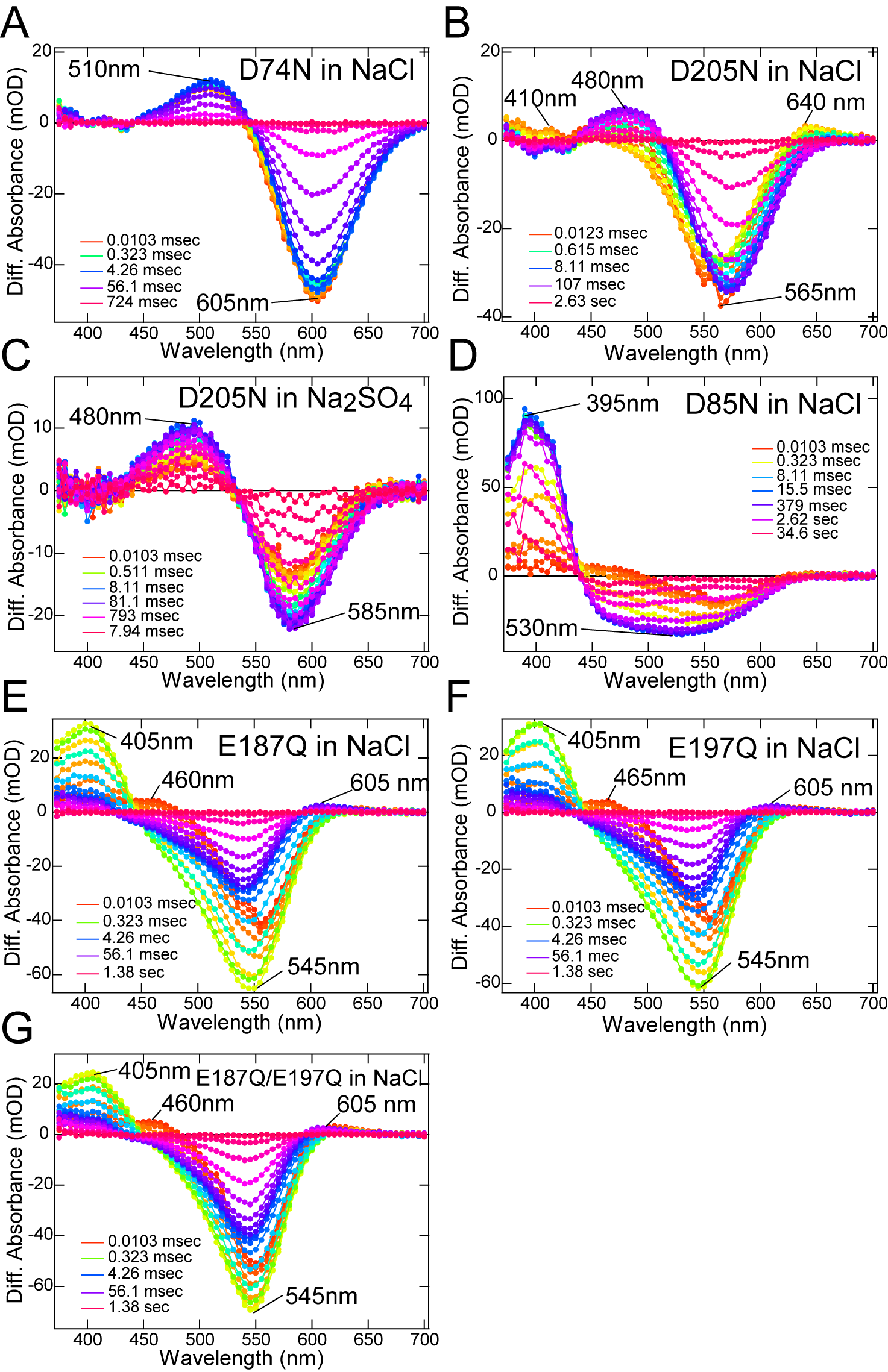


**Figure S5. Photoreaction of wild-type RxR and mutants of RxR.**

Time-resolved difference absorption spectra over a spectral range from 375 to 700 nm of RxR mutants at 25 °C in solutions as noted D74N in NaCl solution (A), D205N in NaCl solution (B), D205N in Na_2_SO_4_ solution (C), D85N in NaCl solution (D), E187Q in NaCl solution (E), E197Q in NaCl solution (F) and E187Q/E197Q in NaCl solution (G).


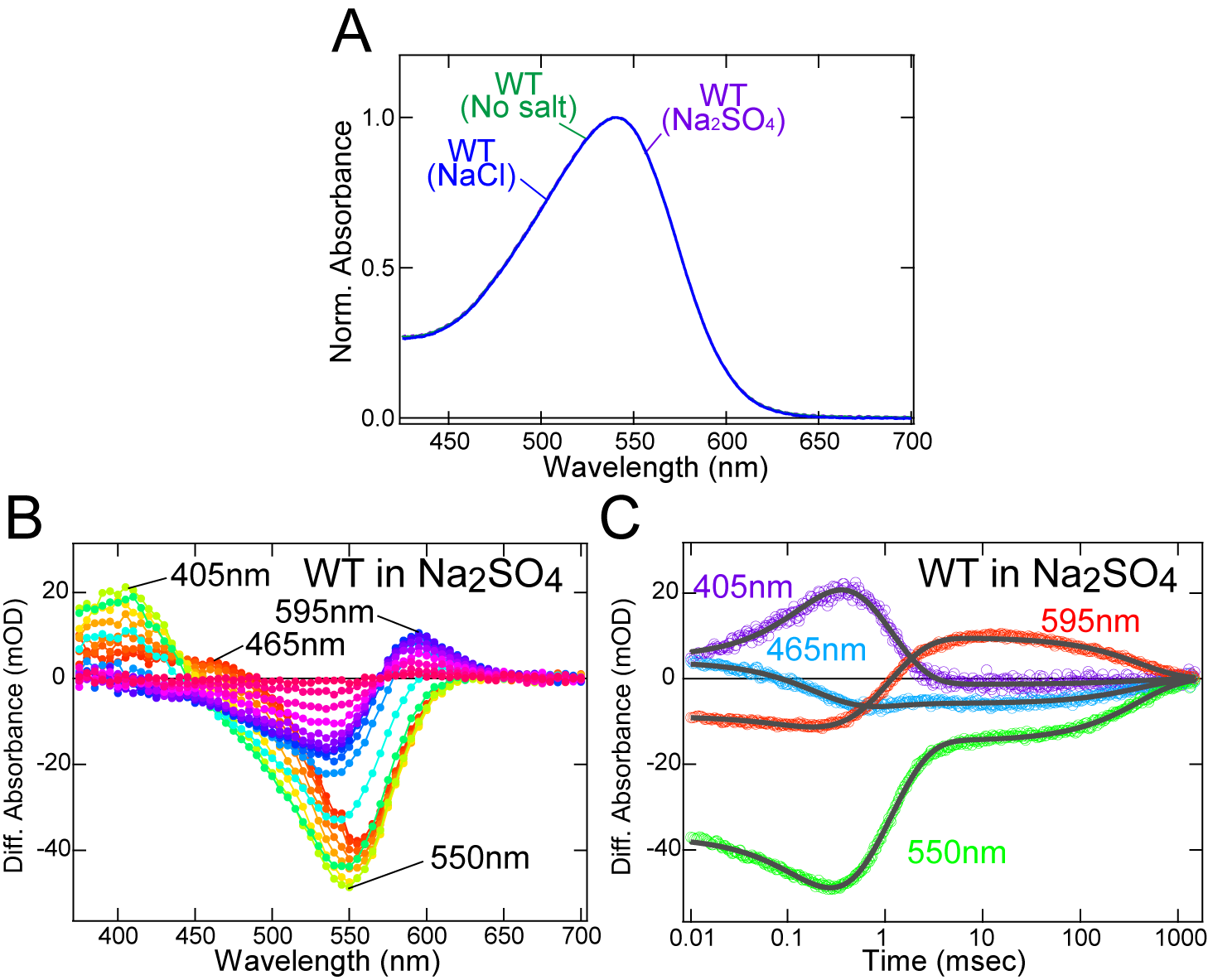


**Figure S6. Absorption spectra and photoreaction of wild-type RxR.**

(A) The absorption spectra of wild-type RxR were measured in NaCl, in Na_2_SO_4_ and in no salt solutions. All spectra were normalized at peak absorbance. (B, C) Flash-photolysis analysis of wild-type RxR in Na_2_SO_4_ solution at 25 °C. (B) Time-resolved difference absorption spectra of wild-type RxR in Na_2_SO_4_ solution over a spectral range from 375 to 700 nm. (C) Time-resolved absorption changes of wild-type RxR in Na_2_SO_4_ solution.


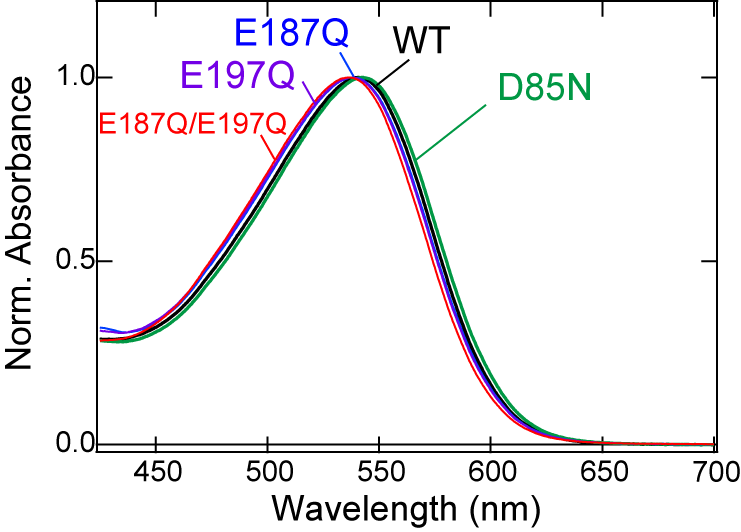


**Figure S7. Absorption spectra of RxR mutants D85N, E187Q, E197Q and E187Q/E197Q.**

The absorption spectra of D85N, E187Q, E197Q and E187Q/E197Q were measured in NaCl solution. All spectra were normalized at peak absorbance.

**References**

1 Kanehara, K., Yoshizawa, S., Tsukamoto, T. & Sudo, Y. A phylogenetically distinctive and extremely heat stable light-driven proton pump from the eubacterium *Rubrobacter xylanophilus* DSM 9941^T^. *Sci. Rep.* **7**, 44427, (2017).

2 Druckmann, S., Ottolenghi, M., Pande, A., Pande, J. & Callender, R. H. Acid-base equilibrium of the Schiff base in bacteriorhodopsin. *Biochemistry* **21**, 4953-4959, (1982).

3 Subramaniam, S., Marti, T. & Khorana, H. G. Protonation state of Asp (Glu)-85 regulates the purple-to-blue transition in bacteriorhodopsin mutants Arg-82→Ala and Asp-85→ Glu: the blue form is inactive in proton translocation. *Proc. Natl. Acad. Sci. U S A* **87**, 1013-1017, (1990).

4 Spassov, V. Z., Luecke, H., Gerwert, K. & Bashford, D. p*K*_a_ calculations suggest storage of an excess proton in a hydrogen-bonded water network in bacteriorhodopsin. *J. Mol. Biol.* **312**, 203-219, (2001).

5 Sampogna, R. V. & Honig, B. Environmental effects on the protonation states of active site residues in bacteriorhodopsin. *Biophys. J.* **66**, 1341-1352, (1994).

6 Saito, K., Kandori, H. & Ishikita, H. Factors that differentiate the H-bond strengths of water near the Schiff bases in bacteriorhodopsin and Anabaena sensory rhodopsin. *J. Biol. Chem.* **287**, 34009-34018, (2012).

7 Edgar, R. C. MUSCLE: multiple sequence alignment with high accuracy and high throughput. *Nucleic. Acids. Res.* **32**, 1792-1797, (2004).
